# Inferring the Origin of Cultivated *Zizania latifolia*, an Aquatic Vegetable of a Plant-Fungus Complex in the Yangtze River Basin

**DOI:** 10.3389/fpls.2019.01406

**Published:** 2019-11-08

**Authors:** Yao Zhao, Zhiping Song, Lan Zhong, Qin Li, Jiakuan Chen, Jun Rong

**Affiliations:** ^1^Jiangxi Province Key Laboratory of Watershed Ecosystem Change and Biodiversity, Center for Watershed Ecology, Institute of Life Science and School of Life Sciences, Nanchang University, Nanchang, China; ^2^The Ministry of Education Key Laboratory for Biodiversity Science and Ecological Engineering, Institute of Biodiversity Science, Fudan University, Shanghai, China; ^3^Institute of Vegetable, Wuhan Academy of Agriculture Science and Technology, Wuhan, China

**Keywords:** domestication, *Zizania latifolia*, *Ustilago esculenta*, genetic structure, approximate Bayesian computation

## Abstract

Crop domestication is one of the essential topics in evolutionary biology. Cultivated *Zizania latifolia*, domesticated as the special form of a plant-fungus (the host *Zizania latifolia* and the endophyte *Ustilago esculenta*) complex, is a popular aquatic vegetable endemic in East Asia. The rapid domestication of cultivated *Z. latifolia* can be traced in the historical literature but still need more evidence. This study focused on deciphering the genetic relationship between wild and cultivated *Z. latifolia*, as well as the corresponding parasitic *U. esculenta*. Twelve microsatellites markers were used to study the genetic variations of 32 wild populations and 135 landraces of *Z. latifolia*. Model simulations based on approximate Bayesian computation (ABC) were then performed to hierarchically infer the population history. We also analyzed the ITS sequences of the smut fungus *U. esculenta* to reveal its genetic structure. Our results indicated a significant genetic divergence between cultivated *Z. latifolia* and its wild ancestors. The wild *Z. latifolia* populations showed significant hierarchical genetic subdivisions, which may be attributed to the joint effect of isolation by distance and hydrological unconnectedness between watersheds. Cultivated *Z. latifolia* was supposedly domesticated once in the low reaches of the Yangtze River. The genetic structure of *U. esculenta* also indicated a single domestication event, and the genetic variations in this fungus might be associated with the diversification of cultivars. These findings provided molecular evidence in accordance with the historical literature that addressed the domestication of cultivated *Z. latifolia* involved adaptive evolution driven by artificial selection in both the plant and fungus.

## Introduction

Cultivated plants are fundamental materials for ensuring the survival and development of human beings (Hajjar and Hodgkin, 2007; Ronald, 2014; FAO, 2019). Through domestication, humans are able to change the morphological and physiological performances of wild plants. With ongoing selective pressure, wild plants would finally adapt to human-derived cultivation environments and provide products that humans demand (Zeder et al., 2006; Gross and Olsen, 2010; Giovannoni, 2018). Plant domestication is regarded as a complex plant-animal co-evolution process, and it is an ideal model to study evolution (Purugganan and Fuller, 2009; Tufto, 2017; Whitehead et al., 2017; Zeder, 2017). As a typical scenario of recent rapid evolution, domestication has demonstrated how a species diverges from its ancestor through artificial selection (Zeder et al., 2006; Purugganan and Fuller, 2009; Diez et al., 2015; Wang et al., 2018).

For a cultivated plant, it is of great importance to decipher the puzzle of its domestication to understand the genetic basis for further improvements. Regardless of the domesticated species we study, there are several questions that need to be answered: what the ancestor is, when and where the domestication occurred, and what happened during the wild-to-cultivated transition (Doebley et al., 2006; Zeder, 2015). Benefitting from cumulative archaeological evidence and the advances of molecular biology, these questions appear to be well resolved for some major crops (Doebley et al., 2006). For example, archaeological and genetic evidence indicated that maize (*Zea mays*) had been domesticated once from its ancestor species teosinte (*Zea mexicana*) in Mexico (reviewed in van Heerwaarden et al., 2011), and common bean (*Phaseolus vulgaris*) had more than two independent domestication events (reviewed in Bitocchi et al., 2012). However, the origins of well-studied crops still may be under debate due to the uncertainty and complexity of historical human activities, such as Asian cultivated rice (*Oryza sativa*) (Ge and Sang, 2011; Huang et al., 2012; Choi and Purugganan, 2018) and a few grain crops generally thought to have originated from the Fertile Crescent (*Hordeum vulgare*, *Triticum aestivum*, and *Avena sativa*) (Fuller et al., 2012; Heun et al., 2012). Moreover, the answers to the domestication puzzles for a given species could also be blurred by post domestication migrations or genetic improvements (eg. cross-regional introduction, diversification of varieties, and hybridizations with wild progenitors) (Burger et al., 2008; Diez et al., 2015). Therefore, cultivated plants may be domesticated in their own way, which should be dissected independently.

The genus *Zizania* belongs to the rice tribe (Poaceae, Oryzeae), and it is an aquatic genus with a discontinuous distribution between eastern Asia and North America (Wen, 1999; Wu et al., 2006). Of the four species in the genus, two are field crops with their seeds harvested for food (the annual *Zizania palustris* native to North America)or enlarged young shoots as vegetable (the perennial *Zizania latifolia* native to Asia) (Oelke, 1993; Xu et al., 2010). Because the young shoots of *Z. latifolia* become swollen, soft, and edible after being infected by the obligatory smut fungus *Ustilago esculenta* and have high nutritional and economic value, it had been domesticated as a vegetable called Jiaobai which is now widely cultivated in the Yangtze River Basin (Guo et al., 2007; Ke, 2008). The cultivated *Z. latifolia* is highly domesticated. The phenotypic differences between wild and cultivated *Z. latifolia* are distinct: the domesticated plant usually has erected plant architecture, broader leaves and shortened internodes; and it could generate much larger swollen shoots containing few black spots (Guo et al., 2007). Once *Z. latifolia* is infected by *U. esculenta*, it loses the ability to undergo sexual reproduction *via* seeds because the fungus hinders the development of the flower primordium. Thus, the breeding of new accessions can only rely on the natural somatic mutants in tillers or rhizomes. In each generation, individuals with good-quality products (swollen shoots) should be screened out for clonal propagation to ensure the stability of agronomic traits of offspring (Guo et al., 2007). The plants that generate flowers or those with too many black spots in the swollen shoots are removed during harvest. Although precautions have been made, unfavorable mutants always occur in the fields, which may be attributed to the high variability of the smut fungus (Guo et al., 2007). To date, there are two main ecotypes including more than 100 local varieties with distinct phenotypic differences. One ecotype is a single-season plant that only can be harvested once each year in the fall (from August to November). This ecotype is a strictly short-day plant that can produce swollen shoots only when the days become short in fall. The other is a double-season crop that is planted in the spring and then can be harvested twice in the fall and the summer thereafter, and it is insensitive to light and endemic to the Yangtze River Basin. The single-season ecotype is taller and has fewer underground rhizomes, lower yield, and a wider distribution relative to the double-season ecotype.

Historical records can help to trace the domestication process of *Z. latifolia* in China (Ke, 2008). The oldest record showed that grains of *Z. latifolia* had been harvested to offer as tribute to the nobles in the Zhou Dynasty (from 771 to 221 BC). Until the Tang Dynasty (618–907 AD), *Z. latifolia* was famous for the tender taste of its grains. During the period between the Zhou and Tang Dynasties, the swollen shoots were rarely reported to be used for food. Only in the low reaches of the Yangtze River was it occasionally eaten by local residents. After the Tang Dynasty, people no longer harvested the grains of *Z. latifolia*. Alternatively, *Z. latifolia* was domesticated to be an aquatic vegetable (Guo et al., 2007; Chen et al., 2012; Chen et al., 2017). Although the utilization history of *Z. latifolia* has provided important clues, several blanks still remain. It was possible that *Z. latifolia* underwent pre-domestication during a long-time use as a food. In the meantime, *U. esculenta* acted as a plant disease during grain production. Then, the plant-fungus complex was domesticated. In another scenario, this vegetable was directly domesticated once or twice after the Tang Dynasty and then generated two main ecotypes with many cultivated varieties. However, these scenarios are just assumptions, and they still lack solid molecular evidence.

Cultivated *Z. latifolia* is the one and only crop that was domesticated in the form of a plant-fungus complex. Undoubtedly, its domestication was associated with the responses of the plant and the fungus to artificial selection. Thus, the cultivated *Z. latifolia* could not only provide a unique case of domestication of a plant-fungus complex but also an ideal model to investigate changes in the genetic interactions of two closely linked species under artificial selective pressures. The plant and the fungus have been independently studied. Wild populations of *Z. latifolia* are widely distributed across eastern China. This plant is of importance as a genetic resource for breeding and forage (Chen et al., 2006). Many studies have been conducted on *Z. latifolia*, including investigation of its phylogeny (Ge et al., 2002; Guo and Ge, 2005; Xu et al., 2010), ecology (Yang et al., 1999), population structure (Xu et al., 2008; Chen et al., 2012), utilization as a tertiary gene pool of rice (Liu et al., 1999), nutritional value (Zhai et al., 2001), cultivar classification, and breeding (Guo et al., 2007). The phylogeny and cytology of *U. esculenta* have been investigated as well (Stoll et al., 2005; Zhang et al., 2012). The genetic interactions between plant and fungus were recently revealed by whole-genome sequencings and comparative transcriptome analyses (Guo et al., 2015; Wang et al., 2017; Ye et al., 2017; Zhang et al., 2017). However, the origin of cultivated *Z. latifolia* has seldom been genetically addressed and remains unknown.

This study focused on dissecting the genetic structure of *Z. latifolia* and the parasitic *U. esculenta*. Twelve microsatellites markers were used to investigate the genetic variations in *Z. latifolia* populations, and model simulations with approximate Bayesian computation (ABC) were then performed to infer the domestication history. The pattern of genetic variations for *U. esculenta* was identified based on ITS sequence analyses. By combining the genetic structures of host and endophyte, the domestication scenario of cultivated *Z. latifolia* could be unveiled.

## Materials and Methods

### Plant Collection

**Figure 1:**
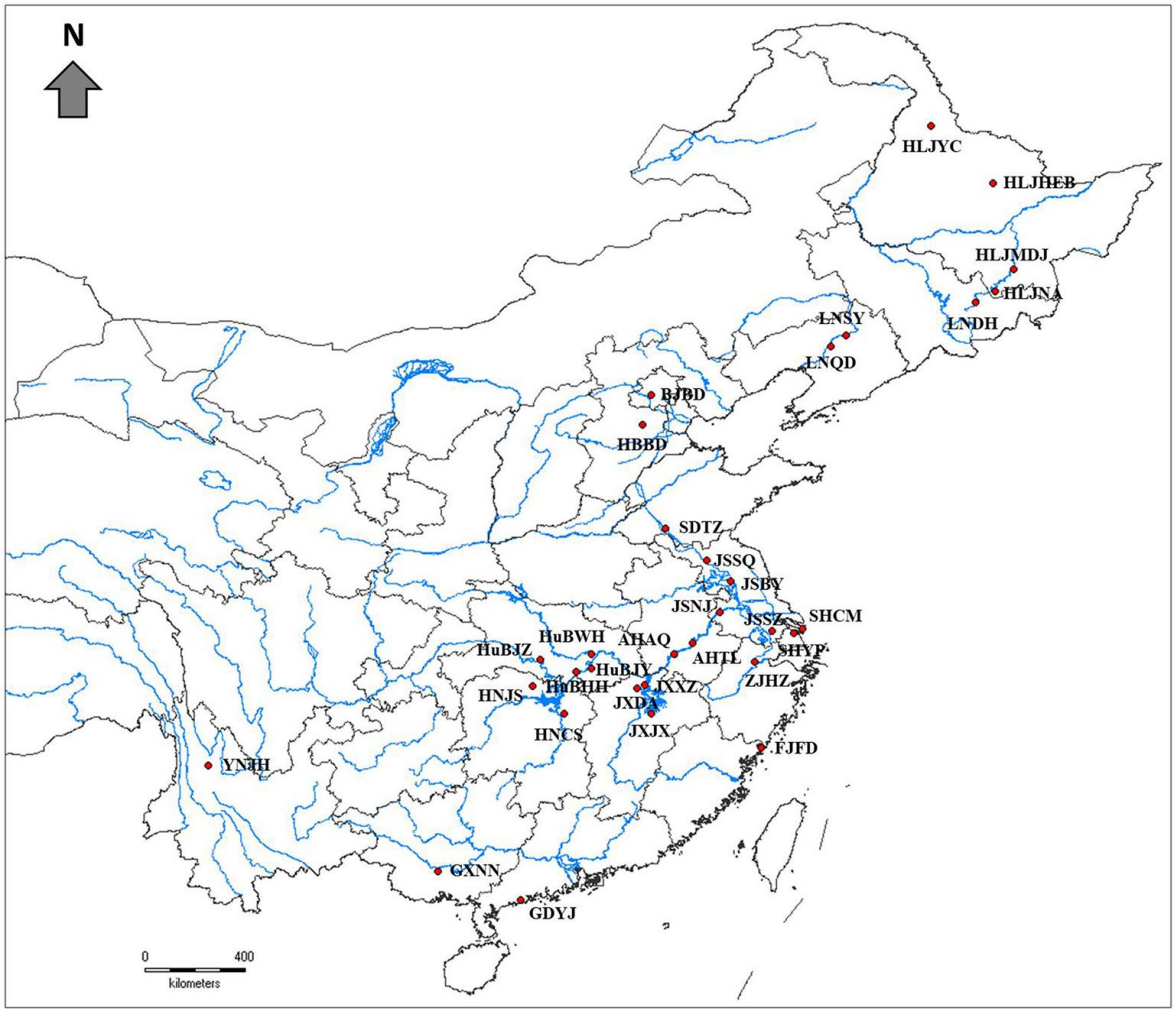
Geographical locations of 32 wild *Zizania latifolia* populations in China (red filled circles).

Thirty-two natural populations of *Z. latifolia* were collected across the species distribution range in China (**Figure 1**, **Table 1**). in each population, the individuals were randomly sampled with an interval of 10 M between plants to reduce the chance of collecting samples from the same genotype. Fresh young leaves were collected from each plant and placed into plastic bags containing silica gel for dehydration. A total of 824 samples of wild *Z. latifolia* were finally obtained. The leaf samples of 135 landraces of cultivated *Z. Latifolia* (the detail information of landraces were attached in **Supplementary Table 2**) were provided by the National Aquatic Vegetable Germplasm Nursery of Chinese Academy of Agriculture Science, Wuhan.

**Table 1:** Parameters of genetic diversity of *Zizania latifolia* populations and cultivars based on 12 microsatellites.

Code	Population	*N*	*A*_r_	*A*_e_	*H*_o_	*H*_e_	*F*	*N*_e_	*Ni*	*Q (> 0.05)*	*Bottleneck*	*Habitat*
1	HLJYC	30	2.909	1.979	0.383	0.383	0.022	226				Pond
2	HLJHEB	6	3.182	2.402	0.273	0.563	**0.450**	152		0.054	S	River
3	HLJMDJ	26	2.727	1.647	0.270	0.310	**0.188**	210				Pond
4	HLJNA	30	3.273	2.078	0.415	0.457	0.069	191			Y	Lake
5	JLDH	42	3.273	1.965	0.346	0.464	**0.275**	305				River
6	LNSY	30	4.545	2.895	0.483	0.585	0.111	233				River
7	LNQD	28	4.818	2.653	0.478	0.539	0.051	276				Stream
8	BJHD	40	2.000	1.407	0.243	0.229	−0.049	256				Stream
9	HBBD	12	3.091	2.177	0.269	0.494	**0.407**	171				Marsh
10	SDTZ	34	3.727	2.140	0.395	0.472	0.108	254				Marsh
11	JSBY	39	5.727	2.893	0.567	0.588	−0.004	312				Lake
12	JSSQ	25	4.000	2.059	0.449	0.425	−0.068	255				River
13	JSNJ	14	2.818	2.116	0.483	0.487	0.013	169			S	Pond
14	JSSZ	30	4.273	1.972	0.524	0.441	−0.102	251				Lake
15	SHYP	18	1.636	1.544	0.533	0.284	−**0.816**	172			Y, S	Stream
16	SHCM	12	2.818	2.538	0.618	0.570	−0.114	150	4	0.330	Y, S	Stream
17	ZJHZ	27	3.455	1.973	0.461	0.438	0.001	192	3	0.114		Marsh
18	AHTL	14	2.091	1.705	0.571	0.371	−**0.468**	208			Y, S	Pond
19	AHAQ	33	4.545	2.493	0.498	0.546	0.058	280				Marsh
20	JXXZ	15	4.364	2.660	0.467	0.511	−0.004	187				Lake
21	JXDA	29	1.818	1.302	0.250	0.167	−**0.170**	210				Stream
22	JXJX	39	3.364	2.264	0.461	0.465	−0.039	304			Y	Stream
23	HuBWH	31	3.545	2.240	0.538	0.505	−0.098	278			Y	Lake
24	HuBJY	27	3.364	2.397	0.477	0.439	−0.133	231				Stream
25	HuBHH	32	4.727	2.545	0.542	0.546	−0.038	297				Marsh
26	HuBJZ	33	4.455	2.632	0.494	0.526	0.030	278				Stream
27	HuNJS	38	3.000	1.994	0.589	0.459	−0.222	273				Pond
28	HuNCS	35	1.455	1.450	0.434	0.229	−**0.918**	120	35	0.994	Y, S	Pond
29	FJFD	14	1.818	1.676	0.539	0.304	−**0.868**	112	14	0.987	Y, S	Stream
30	GDYJ	15	1.636	1.636	0.636	0.329	−**1.000**	150			Y, S	Stream
31	GXNN	15	1.636	1.636	0.636	0.329	−**1.000**	158			Y, S	Stream
32	YNJH	11	2.182	2.013	0.355	0.374	0.023	137	5	0.471	Y, S	Lake
Overall			3.196	2.096	0.459	0.432	−0.135					
SD			1.118	0.434	0.113	0.112	0.383					
	Cultivars	135	2.182	1.519	0.455	0.242	−**0.751**	100	—	0.997	Y	

N, sample size; A_r_, allelic richness; A_e_, number of effective alleles; H_o_, observed heterozygosity; H_e_, expected heterozygosity; F, fixation index; N_e_, the effective population size; Ni, the number of individuals that genetically identical to cultivars; Q, the introgression coefficient; the significant bottleneck effect is labeled by Y (TPM model) and S (Mode shift). The F values that significantly deviated from 0 are shown in bold font.

### Infected Plant Collection and Fungi Isolation

Among the 32 wild populations, we found five individuals infected by the smut fungus *U. esculenta*. A total of 37 strains of *U. esculenta* were isolated from 5 wild accessions and 32 cultivars following the method described by Zhang et al. (2012). These strains were grown on potato dextrose agar (PDA) slant at 26°C and stored in a 10°C incubator.

### DNA Extraction and PCR Assays

Total genomic DNA was extracted following the protocol described by Song et al. (2006). Twelve microsatellites were selected from 100 Asian cultivated rice microsatellites (www.gramene.org) and 16 *Z. latifolia* specific microsatellites (Quan et al., 2009) (**Table S1**). The PCR products were labeled with fluorescent dyes using 5′-tagged forward primers (FAM, JOE, and ROX) and GS350 as an internal size standard labeled with TAMRA. The PCR products were sequenced on an ABI 3730 (Applied Biosystems) automated sequencer. The resulting chromatograms were visualized and analyzed using GENEMAPPER v4.0 software (Applied Biosystems).

The ITS region of smut fungus was amplified using ITS1 and ITS4. PCR products were purified and sequenced on an ABI 3130XL capillary sequencer (Applied Biosystems). All ITS sequences were deposited into GeneBank, with accession numbers MK811211-MK811247.

### Genetic Data Analyses

The frequencies of putative null alleles were estimated using the software FREENA (Chapuis and Estoup, 2007). Null alleles were detected in 34 of 384 population-locus combinations, and the presence of null alleles was not associated with particular populations or loci. We did not find a significant bias in the estimations of *F*_st_ based on the data of all loci compared to the estimate based on corrected genotype data for null alleles (*t*-test, *P* = 0.419). The parameters of population genetic variations were estimated by expected heterozygosity (*H*_e_), observed heterozygosity (*H*_o_) and Wright’s fixation index *F* using GENALEX 6.5 (Peakall and Smouse, 2012). We also calculated the mean effective allele number (*A*_e_) and allelic richness (*A*_r_). Hardy–Weinberg equilibrium departures were tested using exact tests implemented in GENEPOP v4.0 for each population–locus combination. A global test across loci for departures from Hardy–Weinberg equilibrium was conducted using Fisher’s method. Significant deviations were further evaluated using the sequential Bonferroni test.

BOTTLENECK (Piry et al., 1999) was used to screen each population for bottleneck signatures over the last two to four *N*_e_ generations or in a recent period. A significant heterozygote excess or deficit due to a bottleneck was assessed by a two-tailed Wilcoxon signed rank test with 1,000 iterations under the assumption of a stepwise mutation model. At the same time, the distribution of allelic frequencies was drawn to identify whether there had been a mode shift away from an L-shaped distribution, indicating recent population bottlenecks.

MIGRATE (Beerli, 2008) was used to estimate the effective population size *N*_e_. MIGRATE uses coalescent theory to jointly estimate the mutation scaled population size *θ* (4*N*_e_ *µ*) over a long period of time (∼4*N*_e_ generations). Three runs were conducted using MIGRATE. First, two shorter runs (10 short chains of 10,000 sampled, 500 record and three final chains of 100,000, 5,000 recorded) were performed and used to verify that the MCMC estimated the parameters correctly. Then, a final run (10 short chains of 10,000 sampled, 500 recorded and three final chains of 500,000 sampled and 25,000 recorded) was performed, and *θ* values from this final run are reported. The initial run used an estimate of *F*_st_ as a starting parameter to calculate *θ*. Each subsequent run used the ML estimates from the previous run as new starting parameters.

Principal coordinate analysis (PCoA), as implemented in GENALEX 6.5, was conducted to investigate the genetic divergence between cultivated and wild *Z. latifolia* plants. The genetic relationship between the cultivated and wild plants was also estimated using STRUCTURE 2.3.4 (Pritchard et al., 2000) with *K* = 2 and following the method described by Harter et al. (2004). If the proportion of an individual’s genome with ancestry (the introgression coefficient *Q*) from the cultivated group was >50%, we defined that finding as a sign of cultivar ferality.

The degree of population divergence was quantified using *F*_st_ with GENEPOP 4.0. A PCoA was performed with the wild populations only to investigate the pattern of genetic divergence within this species. The genetic structure was further explored using the Bayesian clustering algorithm implemented in STRUCTURE 2.3.4. The program was given no prior information on ancestral populations and run 10 times for each value of *K* ancestral populations, under the admixture model with correlated allele frequencies, using 200,000 Markov chain Monte Carlo iterations and a burn-in of 100,000 iterations. We inferred *K* using the *ad hoc* statistic Δ*K* (Evanno et al., 2005). The resulting matrices of estimated cluster membership coefficients were permuted with CLUMPP (Jakobsson and Rosenberg, 2007). The final matrix for each *K* value was visualized with DISTRUCT (Rosenberg, 2004).

The genetic distance (GD) matrix for all pairs of individuals and populations of wild *Z. latifolia* was estimated by using MSA 3.0 (Dieringer and Schlotterer, 2003). Unrooted neighbor-joining trees were constructed using the PHYLIP package 3.6 with 1,000 bootstraps. The relatedness between individuals and populations was visualized with the software Dendroscope 3.

Isolation by distance (IBD) among the wild populations was tested using the Mantel test implemented in GENALEX 6.5. The significance of IBD values was assessed using 9,999 permutations.

### Inference of the Domestication History of Cultivated *Z. latifolia*

We tested possible models of the demographic history of wild and cultivated accessions to infer the domestication history of cultivated *Z. latifolia* using Approximate Bayesian Computing (ABC) (Beaumont et al., 2002), which allows model choice and parameter estimation without the need to calculate the likelihood function of the models. Data sets are generated under the considered models and are reduced to a set of summary statistics. Simulated data sets with the summary statistics closest to those of the observed data are used to determine the posterior distribution of the parameters and the relative posterior probability of each model. DIYABC v2.0 (Cornuet et al., 2014) was used to compare different models and infer historical parameters. According to the geographical distribution and Bayesian clustering approach implemented in STRUCTURE, the *Z. latifolia* populations could be assigned into two genetic groups, and the southern group showed watersheds-related genetic subdivisions (see the Results section for more details). To reduce the complexity of scenario settings, hierarchical methods were applied following Lombaert et al. (2011). Competing scenarios first were evaluated between the two groups to identify the one with the highest posterior probability and then repeated within the supporting group among subgroups.

We then used an ABC approach to infer demographical parameters for the best scenario. The prior parameters and designs of the scenarios are given in **Table 2** and **Figures 3** and **4**. To construct the reference table, we calculated 10,000,000 simulated data sets for each competing scenario in DIYABC. The posterior probabilities of the competing scenarios were estimated by polychotomous logistic regression based on the 1% of data sets of the simulated reference table, according to the method described in Cornuet et al. (2014).

**Table 2:** Prior distribution of parameters for hierarchical ABC analyses and posterior parameters estimation.

	Prior parameters		Value	Posterior parameters			Quantile 2.5%	Quantile 97.5%
	Parameter	Distribution		Mean	Median	Mode		
Analysis 1 (2 scenarios)								
	*N*s	Uniform	10–20,000	9,180	8,650	7,070	3,330	11,300
	*N*1	Uniform	10–1,000	247	228	199	49.3	319
	*t*1	Uniform	10–2,000	754	697	537	166	1,010
	*t*2	Uniform	10–2,000	971	903	668	250	1,320
Analysis 2 (9 scenarios)								
	*N*e	Uniform	10–20,000	8,040	7,840	7,630	2,660	9,750
	*N*d	Uniform	10–1,000	373	340	269	54.2	505
	*t*1	Uniform	10–2,000	1,600	1,400	1,030	335	2,040
	*t*2	Uniform	10–2,000	1,980	1,710	1,260	508	2,470
	*t*3	Uniform	10–2,000	2,550	1,930	1,330	426	3,290

### The Phylogenetic Analysis of *U. esculenta* ITS Sequences

The ITS sequences were aligned with CLUSTAL W (Thompson et al., 1994) and adjusted manually. The number of polymorphic sites (*S*), haplotype diversity (*H*_d_), and genetic variation measured by average pairwise differences per base pair between sequences (*θ*) (Nei and Li, 1979) and Watterson’s estimates (*θ*_w_) from *S* (Watterson, 1975), were calculated using DNASP version 4.10 (Rozas et al., 2003). Maximum parsimony (MP) searches were performed with 1,000 random taxon addition replicates followed by tree bisection-reconnection branch swapping in PAUP* 4.0b10 (Swofford, 2002). Gaps were treated as missing data. Parsimony bootstrap (PB) for the clades was examined with 1,000 bootstrap replicates using the same options as above.

## Results

### Genetic Variations in *Z. latifolia*

We analyzed plant genotypes for 824 wild and 135 cultivated accessions. Based on the 12 SSR loci, the 959 *Z. latifolia* accessions yielded a total of 138 alleles with 11.46 alleles per locus on average. The mean effective number of alleles (*A*), allelic richness (*A*_r_), observed and expected heterozygosity (*H*_o_ and *H*_e_), and Wright’s fixation index (*F*) are listed in **Table 1**. *H*_e_ ranged from 0.167 (JXDA) to 0.588 (JSBY) among wild populations. The genetic diversity of cultivated accessions (*H*_e_ = 0.242) was much lower than that of wild accessions (*H*_e_ = 0.432). Deviations from Hardy-Weinberg equilibrium were detected in 90 of 384 comparisons after applying the sequential Bonferroni test, which might be due to inbreeding. The Wright’s fixation indices varied greatly among populations (**Table 1**). Several populations (HLJHEB, HLJMDJ, JLDH, HBBD, SHYP, AHTL, JXDA, HuNCS, FJFD, GDYJ, GXNN, and YNJH) displayed signatures of heterozygote excess or deficit.

**Figure 2:**
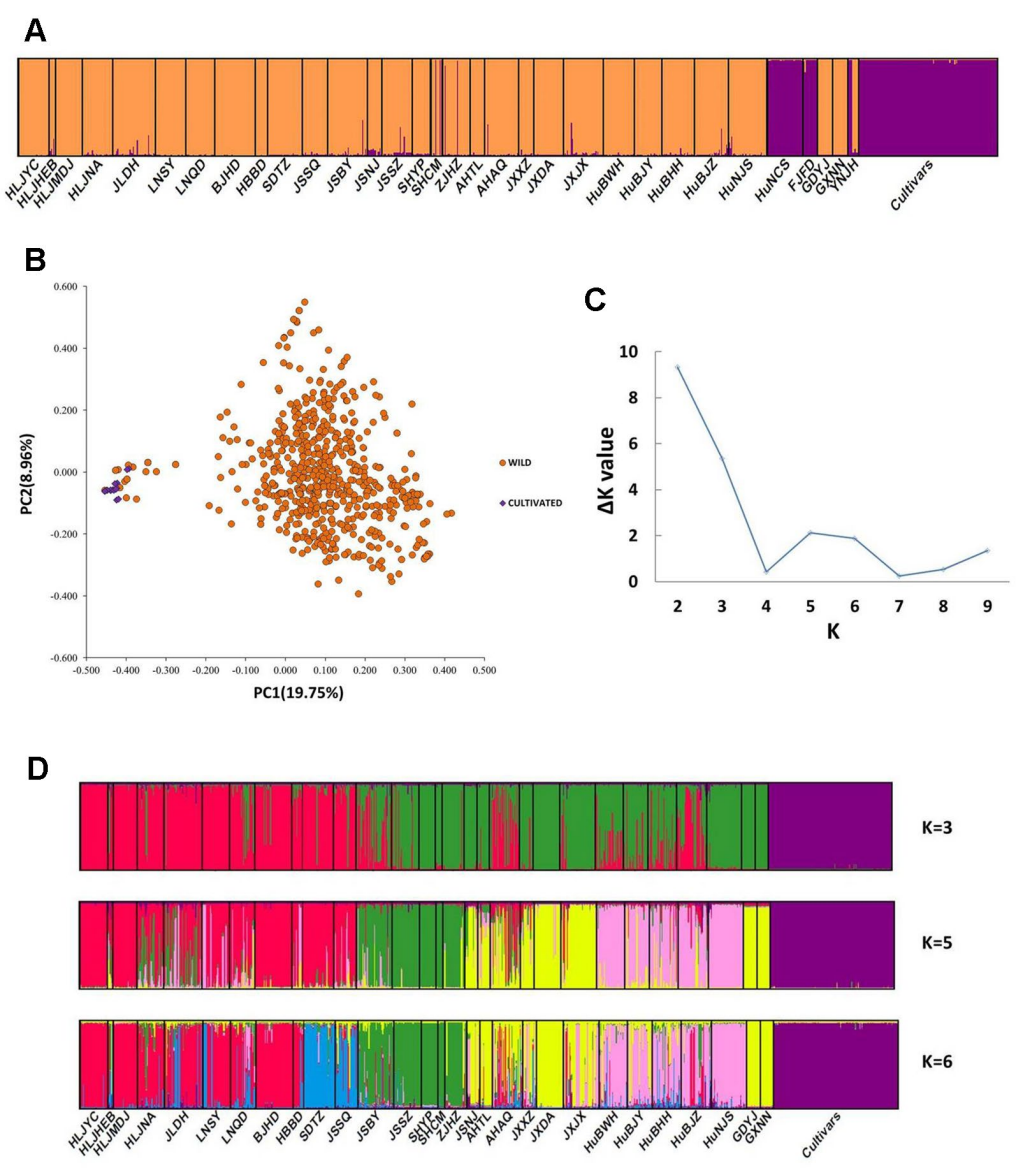
Genetic structure in *Zizania latifolia.* **(A)** The genetic divergence between wild populations and landraces was illustrated by STRUCTURE (*K* = 2). **(B)** Principal Coordinate Analysis (PCoA) of 32 wild populations and cultivated accessions. **(C)** The estimations of Δ*K*. **(D)** Genetic subdivisions in the wild populations of *Z. latifolia* (when *K* = 3, 5 and 6).

The PCoA indicated a distinct genetic divergence between wild and cultivated accessions. However, several putative wild accessions were identified as cultivars. This identification was further supported by the result of STRUCTURE analysis (*K* = 2, **Figures 2A, B**). The Δ*K* analysis indicated that the wild populations of *Z. latifolia* would demonstrate fine genetic structures with an increasing K value, which were shown in **Figures 2C, D**. The HuNCS and FJFD populations were thoroughly composed of plants with cultivar-like genotype. In addition, another 12 accessions from 3 wild populations (SHCM, ZJHZ, and YNJH) were identified as cultivars (**Table 1**). The neighbor-joining tree showed that the cultivars were genetically close to the populations in the lower reaches of the Yangtze River (**Figure S1**).

### Genetic Structure

The effective population size *N*_e_ estimated by MIGRATE ranged from 100 to 312 (*µ* = 10^−4^, **Table 1**). Thirteen of 32 wild populations showed the evidence for historical or recent genetic bottlenecks, suggesting that the effects of genetic drift on these populations were prominent. The cultivars also showed a strong genetic bottleneck due to domestication (**Tables 1** and **2**).

**Figure 3:**
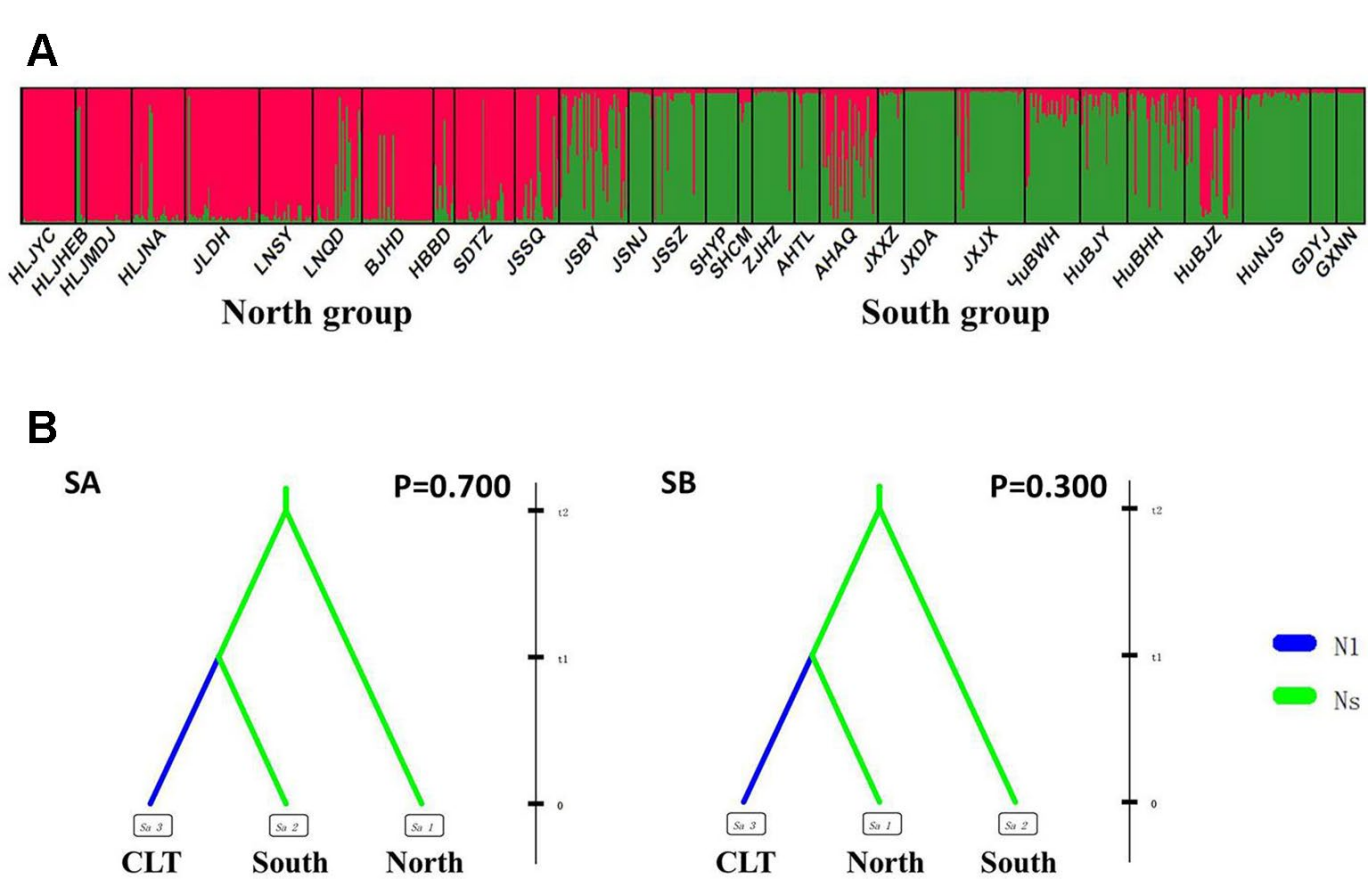
Genetic structure and settings of two competing scenarios in the wild *Zizania latifolia* populations. **(A)** Genetic structure of wild populations illustrated by STRUCTURE. Each vertical bar represents an individual, and each color represents a cluster in the histograms of STRUCTURE. **(B)** Two competing scenarios for ABC simulations. CLT is the cultivated group, South is the south group, and North is the north group. These groups were assumed to be isolated from each other, with no exchange of migrants. In Scenario A (SA), the southern group was separated from the northern group at time scale t2, with no changes in effective population size; at time scale t1, the cultivated group was then separated from the southern group and went through a bottleneck with an effective population size change from *N*s to *N*1.

**Figure 4:**
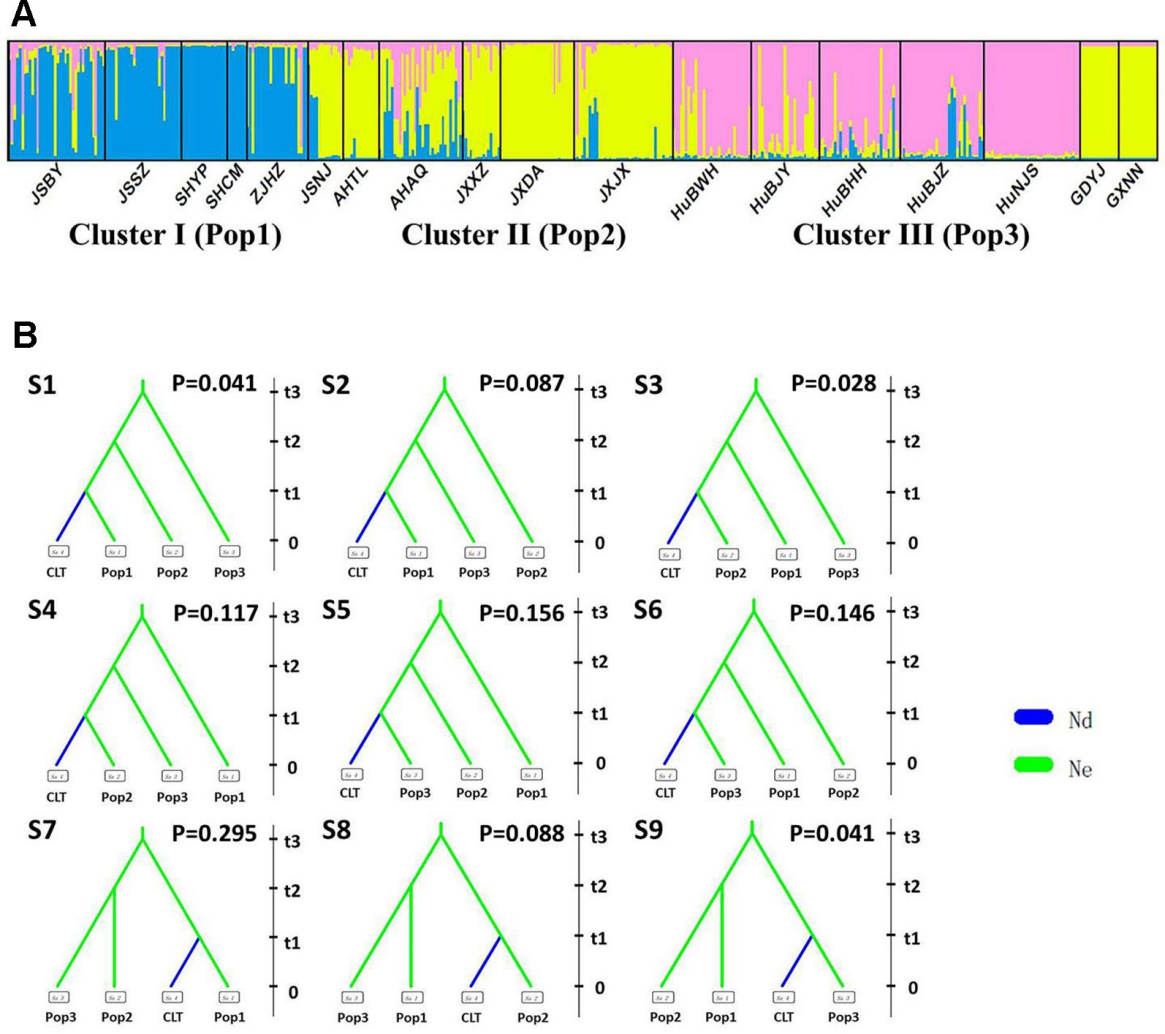
Genetic structure and settings of nine competing scenarios in the southern genetic group of wild *Zizania latifolia*. **(A)** The subtle genetic structure in the southern genetic group of *Z. latifolia* revealed by STRUCTURE. Each vertical bar represents an individual, and each color represents a cluster in the histograms of STRUCTURE. **(B)** 9 competing scenarios for ABC simulations inferring the origin of cultivars in the south group of wild *Z. latifolia* populations. CLT is the cultivated group, Pop1 is the Hongze-Tai lake group, Pop2 is the Poyang lake group, and Pop4 is the Dongting lake group.

After excluding the individuals with cultivar-like genotype (SHCM, ZJHZ, HuNCS, YNJH, and FJFD), *F*-statistics revealed that the wild populations of *Z. latifolia* were genetically divergent (*F*_st_ = 0.320, **Table S1**). The Mantel test suggested a weak but significant sign of isolation by distance among *Z. latifolia* populations (**Figure S2**, *r* = 0.21, *P* < 0.05). The outcomes of a Bayesian assignment with STRUCTURE indicated that *K* = 2 is the best for describing the actual genetic structure of wild populations (**Figure 3A**). One group included the northern populations in the north and Northeast Plain of China, and the remaining southern populations formed another group mainly located in the Yangtze River Basin. However, the PCoA showed no distinct genetic divergence among wild populations, and the first three principal components described 28.6% of the total variances. In addition, the Bayesian assignment of the southern populations revealed watershed-related genetic subdivisions in the Yangtze River Basin: the Hongzhe-Tai Lake region (Cluster I), the Poyang Lake region (Cluster II) and the Dongting Lake region (Cluster III) (**Figure 4A**). Two southern populations located in the Pearl River Basin (GDYJ and GXNN) showed a close ancestry with the populations in the Poyang Lake region cluster (**Figure 4A**).

In particular, we tested the genetic divergence between two ecotypes of cultivars. By using the software STRUCTURE, we found no significant genetic divergence with *K* = 2. Instead, the PCoA revealed the genetic differences between ecotypes (**Figure S3**). The microsatellite marker ZM24 could be an effective molecular marker to distinguish two ecotypes of cultivars.

### Modeling the Demography of *Z. latifolia* Domestication

Following our hierarchical ABC strategy, we first compared the two predesigned scenarios in Analysis 1. Then the posterior parameters of the best-supported scenario were estimated, and the model checking was accessed between the posterior data set and observed data (**Table 2** and **Figure S4A**). Scenario A had a higher posterior probability (0.700) (**Figure 3B**), and it indicated that the cultivars diverged from the group of southern populations. In Analysis 2, 9 competing scenarios were further investigated (**Table 2** and **Figure S4B**). Scenario 7 showed the highest posterior probability (0.295), which indicated that the Hongze-Tai Lake region cluster firstly diverged from the other clusters in the southern group 1930 generations (t3) ago. The Poyang Lake region cluster and Dongting Lake cluster then diverged 1,710 generations (t2) ago. The cultivars were single-domesticated in the Hongze-Tai Lake region 1,400 generations (t1) ago (**Figure 4B** and **Table 2**).

### Sequence Variation and Phylogenetic Analysis

A total of 37 strains of *U. esculenta*, including 5 wild and 32 cultivated strains, were sequenced. The total length of the aligned sequences was 712 bp. Twenty-seven polymorphic sites were observed. Thirty-one haplotypes were found in the wild and cultivated accessions, and the haplotype diversity (*H*_d_) was 0.991. The nucleotide diversity (*θ*) was 0.01, and *θ*_w_ was 0.01.

**Figure 5:**
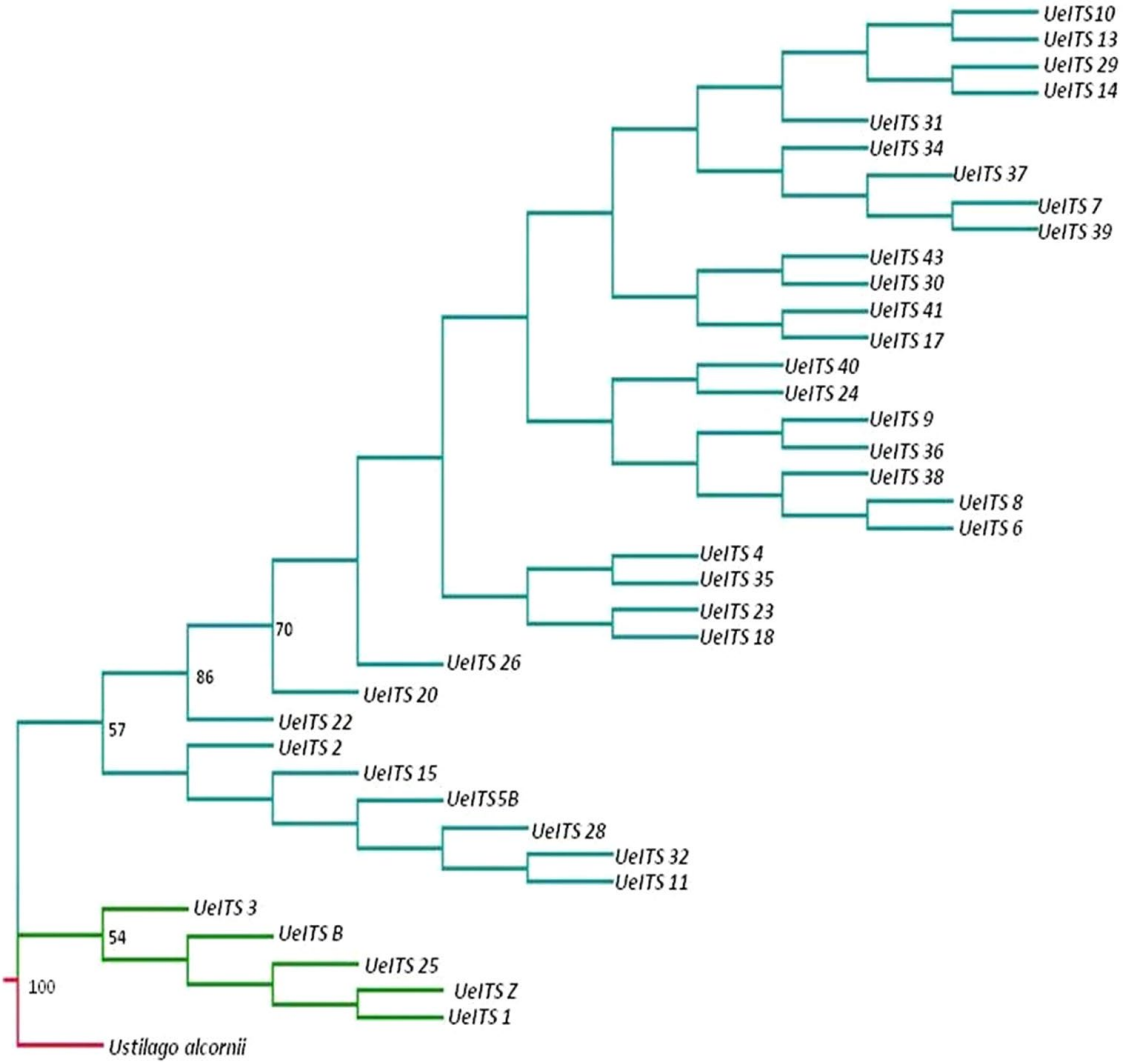
Phylogeny tree (maximum parsimony, MP) for the 37 accessions (wild accessions in green and cultivated accessions in cyan) of *Ustilago esculenta*, *Ustilago alcornii* is the outgroup (in red). Bootstrap values calculated over 1,000 replications are given as percentages (only values > 50% were shown).

An ITS sequence of *U. alcornii* was downloaded from GeneBank and used as the outgroup taxon. Another ITS sequence of *U. esculenta* (Genebank accession number: JX219372.1) was used as the control. The MP tree is shown in **Figure 5**. The structure of the MP tree showed significant divergence between the wild fungi and those from cultivars, indicating a single domestication event.

## Discussion

The domestication of cultivated *Z. latifolia* is a typical example that can demonstrate how a plant-fungus complex rapidly evolved under artificial selections. Our results showed that there was prominent genetic divergence between wild populations and cultivated accessions after long-time cultivation. A single domestication event occurred in the lower reaches of the Yangtze River at the Tang Dynasty (approximately 1,400 years ago), then this vegetable was constantly cultivated and bred. The genetic divergence of the plant may be responsible for the differences between the ecotypes of cultivars, and the genetic variations in *U. esculenta* are likely associated with the diversification of cultivated varieties.

### The Possible Domestication Process of *Z. latifolia*

There are distinct phenotypic differences between wild and cultivated *Z. latifolia*. Compared with its wild ancestor, the cultivated plant usually has an erected plant architecture, broader leaves, and shortened internodes (Guo et al., 2007). The cultivated plants were reported to be vigorous in photosynthetic efficiency (Yan et al., 2013). These morphological and physiological changes together constituted the Domestication Syndrome of cultivated *Z. latifolia*, which would be referenced in distinguishing wild and cultivated plants.

We found significant genetic divergence between wild and cultivated *Z. latifolia* (**Figures 2A, B**). The extent of genetic differentiation between wild and cultivated plants (*F*_st_ = 0.288) was even greater than the differentiation among wild populations (*F*_st_ = 0.175). This phenomenon is usually observed in highly domesticated species such as wheat, rice or maize (Heun et al., 1997; Harter et al., 2004; Zeder et al., 2006; Zhao et al., 2013). Unlike the annual grain crops, the breeding of *Z. latifolia* relies on asexual reproduction. By utilizing natural mutants that occur in tillers or rhizomes, cultivated *Z. latifolia* was able to generate more than 100 varieties. Theoretically, when compared with annual crops bred *via* sexual reproduction, the evolutionary rate of cultivated *Z. latifolia* is relatively low. The low evolutionary rate and short domestication time (<2,000 years) should result in low genetic divergence between wild and cultivated plants, which is contrary to our finding. The unexpected high genetic divergence between wild and cultivated plants could also be attributed to the breeding system of cultivated *Z. latifolia*. Because cultivated *Z. latifolia* would occasionally degenerate to the wild phenotype, farmers had to screen out the elite plants as genets every year. Although the clonal reproduction of cultivated *Z. latifolia* greatly restricted new mutants stemmed from sexual propagation and blocked gene flows from wild accessions to cultivars, the annually strong artificial selection would somehow accumulate mutants, inducing significant genetic divergence between wild and cultivated plants.

Previous studies had inferred that the cultivated *Z. latifolia* may be domesticated in the Yangtze River Basin, but they did not provide any convincing evidence. (Xu et al., 2008; Xu et al., 2015). Using microsatellite markers and model inference, our study located the origin site of cultivated *Z. latifolia* in the areas around Hongze Lake and Tai Lake (**Figure 4**). By ABC modeling, the cultivated *Z. latifolia* diverged from ancestor populations 1,400 generations ago. Given 1 year per generation, the domestication event occurred 1,400 years ago at the Tang Dynasty. Furthermore, the genetic relationship of fungi hinted that the single-season ecotype might have been domesticated first, and then the double-season ecotype was bred from the single-season ecotype. These results were in accordance with the historical records (Ke, 2008).

Based on current molecular data, we could not be sure whether *Z. latifolia* underwent historical pre-domestication as a food crop. There is no historical record or remained landraces that can support the existence of pre-domestication. We never found the plants with any domestication traits similar to cereal crops in the wild, too. However, the vegetable cultivars of *Z. latifolia* could escape to natural habitats. We found typical escaped individuals in five provinces that were the main production areas of cultivated *Z. latifolia* (**Table 1**). Cultivated plants that cannot survive on their own in natural habitats often have maladaptive domestication traits (Campbell and Snow, 2009). Cultivated *Z. latifolia* has lost the ability to undergo sexual reproduction, which would be unfavorable for persistence in the wild. Alternatively, feral *Z. latifolia* relies on asexual growth to reproduce in natural habitats. In fact, it often occupies the shores of ponds or streams near fields by clonal propagation *via* underground rhizomes and detached shoots, just as we had noticed in FJFD and HuNCS. Moreover, without pollen flow, it seems unlikely that wild populations would be introgressed by feral cultivars.

### Genetic Diversity and Population Structure of Wild *Z. latifolia*

Xu et al. (2015) had characterized the genetic diversity of the four species in the genus *Zizania* using 3 cross-specific amplified microsatellite markers. They found that *Z. latifolia* showed a relatively low genetic diversity relative to its annual relative *Z. palustris* in North America (*H*_e_ = 0.374 vs *H*_e_ = 0.630). However, they might have underestimated the genetic diversity of both species due to insufficient genetic markers. In contrast, another study investigated the genetic variations of *Z. latifolia* restricted to the middle reaches of the Yangtze River by using 10 microsatellites and found a high level of genetic diversity (*H*_e_ = 0.532) (Chen et al., 2012). In our study, a relatively high genetic diversity (*H*_e_ = 0.497) was detected.

Most of the fixation indices (*F*) for the high-latitude populations were significantly greater than 0 (**Table 1**), indicating a heterozygous deficit that could be attributed to inbreeding or outbreeding among ramets. However, several populations in low latitudes were composed by a few dominant heterozygous genotypes, which resulted in negative *F* values. This phenomenon might be attributed to local adaption or a rapid population expansion based on a few heterozygous ancients *via* clonal growth. For example, 10 years ago, a few ancestors floating down from upstream colonized and formed the population JXDA (Zhao et al., field surveys). Previous phylogeographic analysis suggested that the genus *Zizania* originated in North America and then dispersed into eastern Asia *via* the Bering land bridge during the Tertiary (Xu et al., 2010). Hewitt (2004) had proposed that the range-wide patterns of population genetic diversity are usually shaped by past climate-driven range dynamic. In support of this view, the genealogical pattern based on the nuclear Adh1a gene of *Z. latifolia* indicated that high-latitude populations harbored more abundant haplotypes (Xu et al., 2008). However, in our study, the populations in high latitudes (the northern group, *H*_e_ = 0.447) did not show a higher genetic diversity than those in low latitudes (the southern group, *H*_e_ = 0.444).

Our results and previous studies all indicated strong genetic differences among populations (**Figure 2**). The Mantel test further revealed the effect of isolation by distance (IBD, **Figure S2**). IBD is expected to be the primary factor leading to a high level of genetic divergence, due to limited gene flow and the effects of genetic drift (Slatkin, 1987). This process was supported by our study on wild *Z. latifolia* populations. The aquatic habitat *of Z. latifolia* is discrete and patchy. These isolated wetlands restrict migration between populations, and as a consequence, aquatic-living plants usually exhibit a high level of genetic divergence among populations (Barrett et al., 1993). The dispersal of seeds or detached shoots by water flow is unpredictable, and these are unlikely to disperse *via* water currents between spatially highly isolated populations. In wind-pollinated species, the pollen can be carried for long distances, but most effective pollination occurs at a local scale (Willson, 1983). Nevertheless, the isolation between wetlands might be counteracted by seasonal flooding, resulting in a temporal connection within a water system. The genetic structure of *Z. latifolia* provided evidence that supported the effects of hydrological connectivity in shaping the genetic structure of aquatic plant species. Isolation by geographical barriers and genetic similarity along rivers was found in regional populations of *Z. latifolia* (Chen et al., 2012; Chen et al., 2017). The effects of hydrological unconnectedness on genetic relationship of populations within a water system were also reported in another emergent macrophyte, *Oryza rufipogon* (Wang et al., 2008).

The genetic assignments and morphological performances of two populations (GDYJ and GXNN) at the leading-edge seemed to be rather special (**Table 1** and **Figure 2D**). On one hand, these populations are cultivated-like in phenotype, but they showed no sign of *U. esculenta* infection; on the other hand, they are genetically different from cultivated *Z. latifolia*, and have heterozygous genotypes. Their small population size and low diversity might be attributed to the “Founder effect.” Bayesian assignment showed they could be grouped to the Poyang Lake region cluster, implying a migration through the geographical barrier (Nanling Mountains).

### The Association of Plant and Fungus Under Artificial Selection

As an obligate parasitic fungus, *U. esculenta* is harmful to its host under natural conditions. The fungus not only inhibits the emergence of inflorescence but also requires a large amount of carbohydrates to form the swollen shoot attractive to herbivorous insects. *U. esculenta* infection is not very common in wild populations, as we have only collected 5 samples across 32 range-wide populations. In our common garden trials, the fitness of infected wild plants is significantly lower than that of healthy ones (Zhao et al., data not shown), which implies a weak competitive ability of the infected plants.

The Red Queen hypothesis proposed that the parasite and host should keep on adapting and evolving to gain reproductive advantages (Van Valen, 1973). The antagonistic co-evolution between species would finally be terminated either by the extinction of one species or the emergence of symbiosis. The domestication of infected *Z. latifolia* seemed to accelerate the process of achieving symbiosis. On the one hand, the persistent selection force of mankind delayed the development of the fungus and extended the mycolic stage (Zhang et al., 2017). On the other hand, humans gradually screened out plants with the genotype that adapted to cultivated conditions (Yan et al., 2013). Moreover, with the efforts exerted by mankind, the distribution range of this plant-fungus complex was largely expanded.

We detected minor but significant genetic differentiation between the hosts of two ecotypes of cultivated *Z. latifolia* (**Figure S3**), which was consistent with the pattern revealed by the sequences of Adh1 (Xu et al., 2008). However, the ITS sequences of *U. esculenta* strains from cultivated Z. latifolia showed unexpectedly high levels of diversity compared with a previous study (You et al., 2011).There might be inconsistent evolutionary rates in the parasite and host. The evolution of the host determines the responses to photoperiod and temperature which resulted in the differences between two ecotypes in cultivated *Z. latifolia* (Guo et al., 2015), and the faster evolutionary rate of the parasite may lead to the diversification of varieties (**Figure 5**, also see Ye et al., 2017).

Currently, it is still difficult to dissect the relative contributions from the host and parasite in shaping Domestication Syndrome. We could only infer the changes in morphological traits during the domestication process based on the phylogeny of cultivated *U. esculenta* (**Figure 5**). First, the single-season ecotype was domesticated from a wild plant-fungus complex. This ecotype retained a few primary traits, such as light sensitivity, long internodes, and small swollen shoot. Then, artificial selections on one of the single-season varieties led to the emergence of the double-season ecotype, which was insensitive to light and could be harvested twice. The double-season ecotype was more advanced than the single-season ecotype, but it could only be cultivated in a restricted region. There is an urgent need to improve the fitness of the double-season ecotype.

## Data Availability Statement

All datasets for this study are included in the article/**Supplementary Material**.

## Author Contributions

YZ, ZS, JC and JR conceived the idea and designed the research project. YZ, QL and LZ collected the data. YZ performed the data analysis. YZ drafted the initial manuscript with contribution from JR and ZS. All the authors contributed critically to the discussion and edited the manuscript before submission.

## Funding

This study was supported by the National Natural Science Foundation of China (31600293) and the China Postdoctoral Science Foundation Grant (2015M571483).

## Conflict of Interest

The authors declare that the research was conducted in the absence of any commercial or financial relationships that could be construed as a potential conflict of interest.
