# Supplementary material for: Inferring the Origin of Cultivated Zizania latifolia, an Aquatic Vegetable of a Plant-Fungus Complex in the Yangtze River Basin
Source: Front Plant Sci. 2019 Nov 8;10:1406. doi: 10.3389/fpls.2019.01406 (PMC6856052; doi:10.3389/fpls.2019.01406)
Supplement: Supplementary file 1 [file Image_1.pdf]

(a)

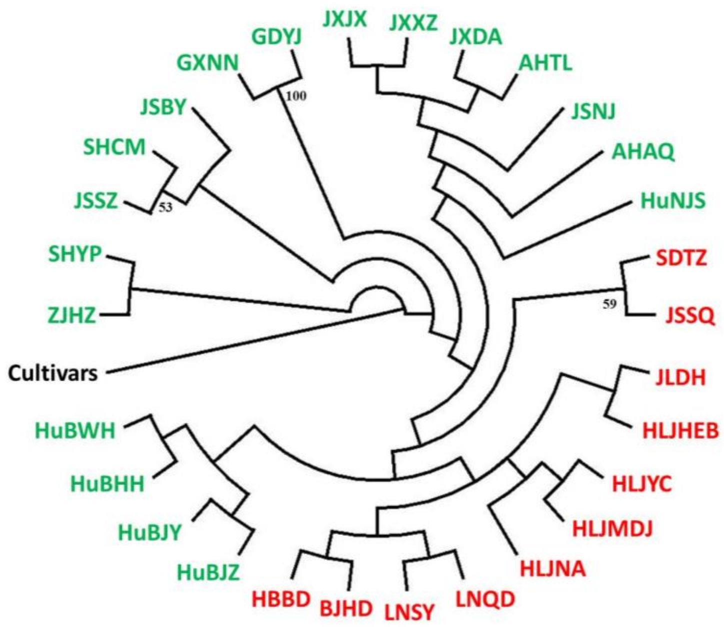

(b)

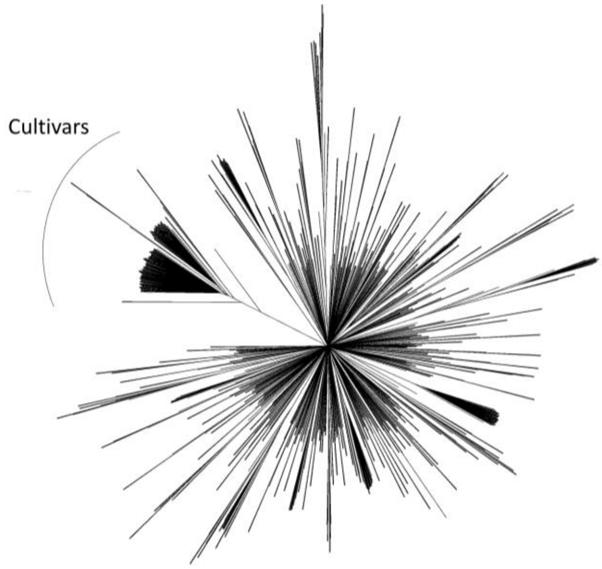

Fig. S1 Unrooted Neighbor-Joining trees for *Zizania latifolia* based on Nei's genetic distance: a, the population based NJ- tree, populations assigned to the north group were shown in red and south group in green, bootstrap values calculated over 1,000 replications are given as percentages (only values > 50% were shown); b, the individual-based NJ tree, the cultivated accessions were illustrated.
