# Supplementary material for: Inferring the Origin of Cultivated Zizania latifolia, an Aquatic Vegetable of a Plant-Fungus Complex in the Yangtze River Basin
Source: Front Plant Sci. 2019 Nov 8;10:1406. doi: 10.3389/fpls.2019.01406 (PMC6856052; doi:10.3389/fpls.2019.01406)
Supplement: Supplementary file 2 [file Image_2.pdf]

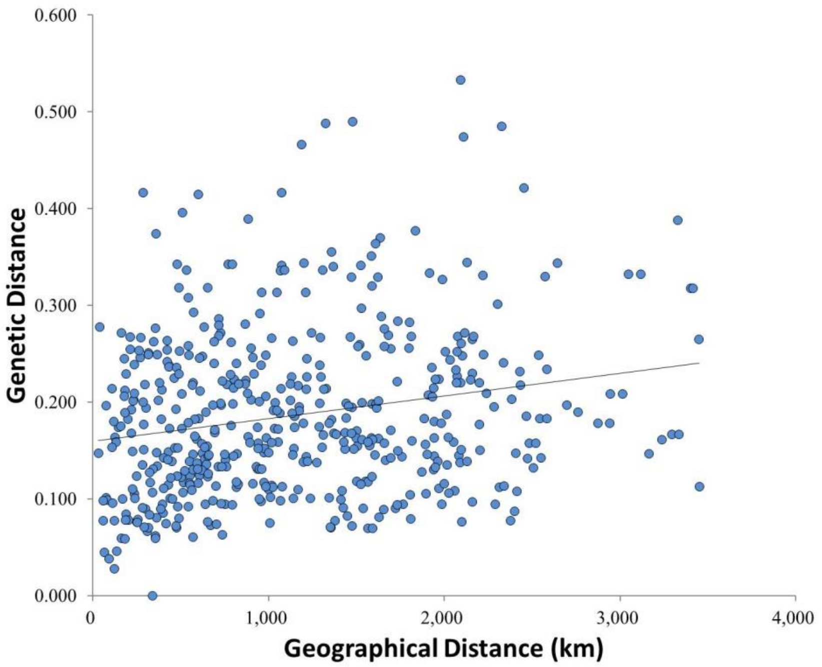

Fig. S2 A scatterplot between genetic distance and geographic distance for *Zizania latifolia* populations. Genetic distance is represented by pairwise $F_{st}/(1–F_{st})$ among populations, which is correlated with the geographic distance between pairwise populations. The Pearson’s regression line overlays the scatterplot (Mantel-test, r = 0.21, $P < 0.05$).
