# Supplementary material for: Inferring the Origin of Cultivated Zizania latifolia, an Aquatic Vegetable of a Plant-Fungus Complex in the Yangtze River Basin
Source: Front Plant Sci. 2019 Nov 8;10:1406. doi: 10.3389/fpls.2019.01406 (PMC6856052; doi:10.3389/fpls.2019.01406)
Supplement: Supplementary file 3 [file Image_3.pdf]

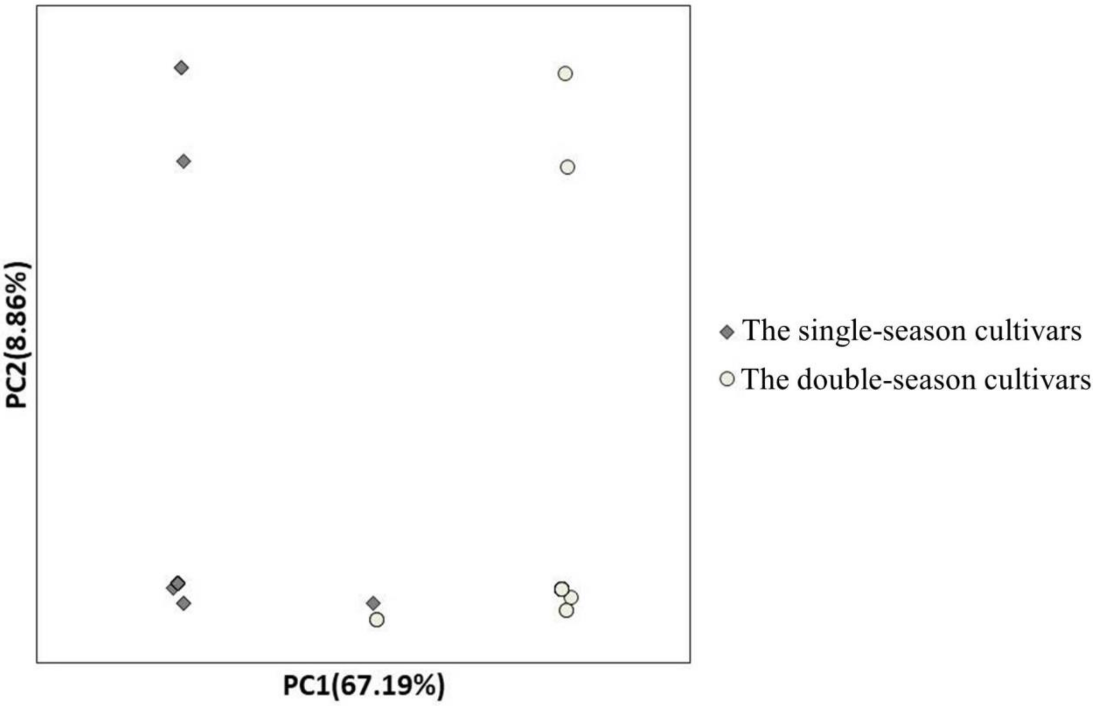

Fig. S3 Principal-Coordinate-Analysis (PCoA) of cultivated *Zizania latifolia* accessions. The two ecotypes were divergent (the filled spades: the single-season cultivars; the hollowed circles: the double season cultivars).
