# Supplementary material for: Inferring the Origin of Cultivated Zizania latifolia, an Aquatic Vegetable of a Plant-Fungus Complex in the Yangtze River Basin
Source: Front Plant Sci. 2019 Nov 8;10:1406. doi: 10.3389/fpls.2019.01406 (PMC6856052; doi:10.3389/fpls.2019.01406)
Supplement: Supplementary file 4 [file Image_4.pdf]

(a)

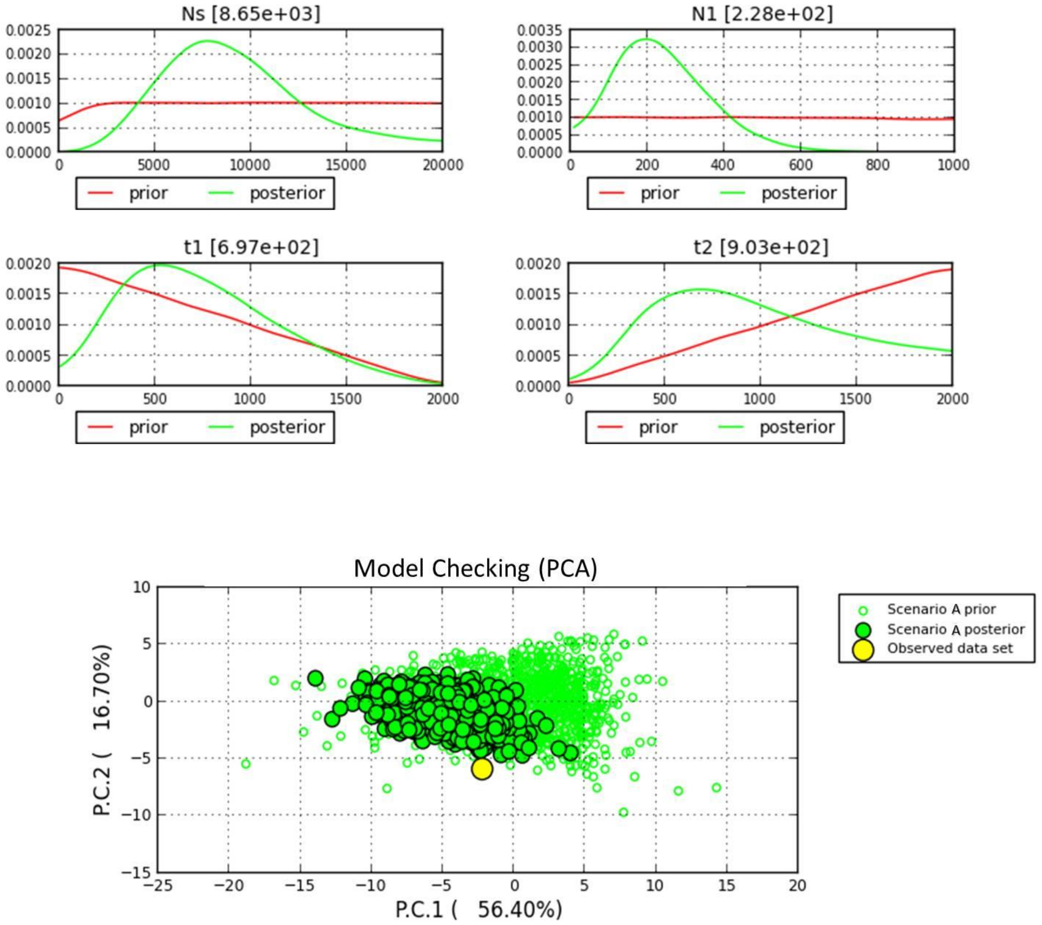

Ns [8.65e+03]
prior
posterior
N1 [2.28e+02]
prior
posterior
t1 [6.97e+02]
prior
posterior
t2 [9.03e+02]
prior
posterior
Model Checking (PCA)
Scenario A prior
Scenario A posterior
Observed data set
P.C.2 (16.70%)
P.C.1 (56.40%)

(b)

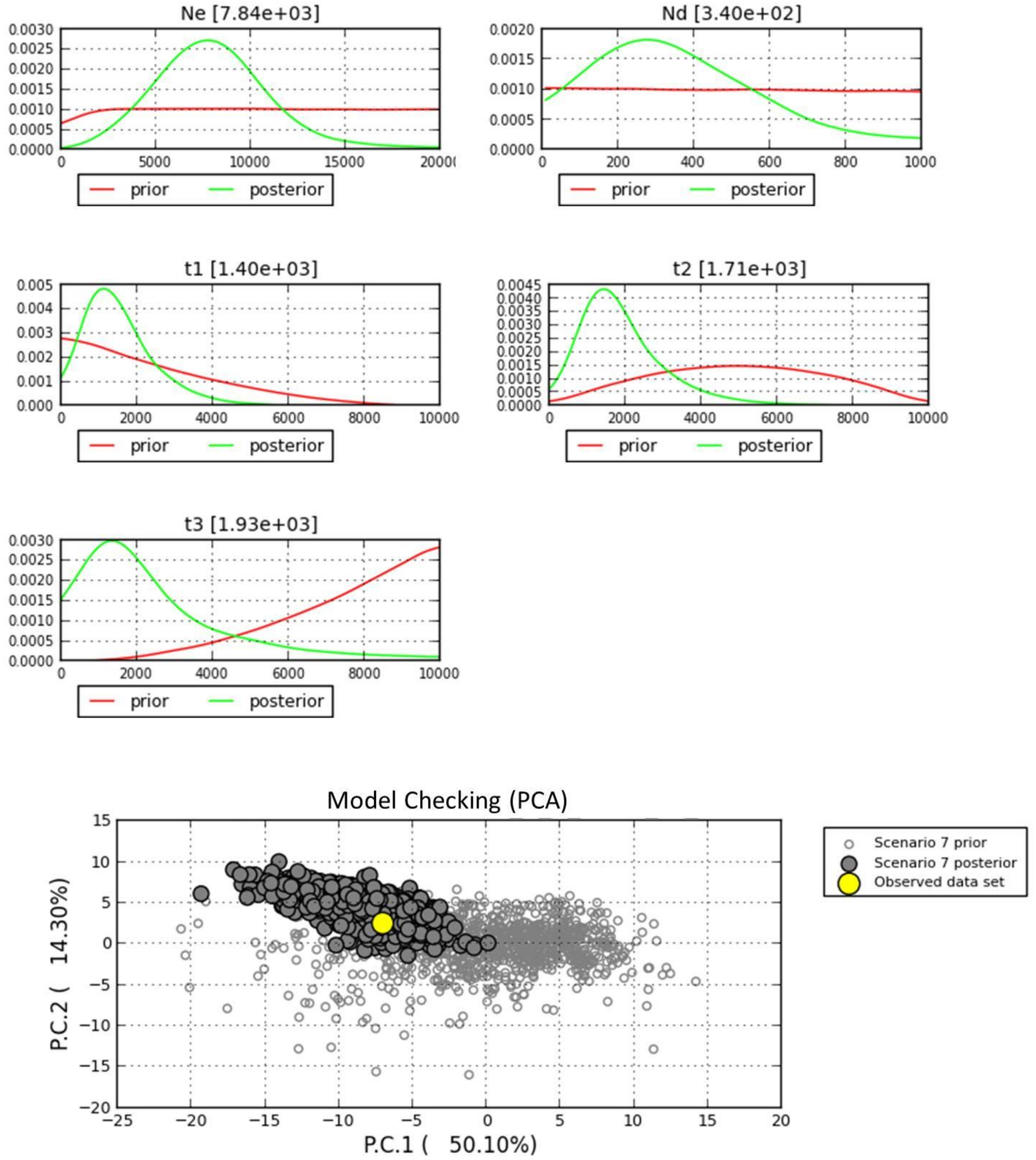

Fig. S4 The prior and posterior distributions of parameters and the Principal Component Analysis (PCA) of model checking in hierarchical ABC analyses. a, the diagrams of parameters (*Ns*, *N1*, *t1*, *t2*) and PCA plots for scenario A; b, the diagrams of parameters (*Ne*, *Nd*, *t1*, *t2*, *t3*) and PCA plots for scenario 7.
