# Supplementary material for: Inferring the Origin of Cultivated Zizania latifolia, an Aquatic Vegetable of a Plant-Fungus Complex in the Yangtze River Basin
Source: Front Plant Sci. 2019 Nov 8;10:1406. doi: 10.3389/fpls.2019.01406 (PMC6856052; doi:10.3389/fpls.2019.01406)
Supplement: Supplementary file 5 [file Table_1.pdf]

Table S1 The parameters of genetic diversity and *F*-statistics for 12 microsatellites

	N	A_e	H_o	H_e	F_{st}
**ZM11**	25	3.167	0.675	0.657	0.298
**ZM16**	16	2.966	0.586	0.578	0.325
**ZM40**	7	1.899	0.382	0.388	0.307
**ZM24**	13	2.886	0.689	0.604	0.292
**ZM25**	8	1.892	0.383	0.389	0.391
**ZM35**	10	1.853	0.427	0.392	0.420
**ZM44**	4	2.087	0.931	0.516	0.075
**ZM26**	4	1.550	0.322	0.307	0.329
**ZM36**	20	2.523	0.241	0.494	0.449
**RM6876**	9	2.090	0.595	0.492	0.240
**ZM28**	19	1.794	0.272	0.334	0.444
**ZM30**	5	1.327	0.216	0.195	0.251
**Mean**	11.455	2.079	0.459	0.426	0.320
**SE**	1.744	0.156	0.066	0.037	0.033

N, number of alleles; A_e, effective number of alleles; H_o, observed heterozygosity; H_e, expected heterozygosity; F_{st}, *F*-statistics.
